# Bovine Meat and Milk Factor-like Sequences Are Frequently Detected in Renal Cell Carcinoma Tissues

**DOI:** 10.3390/cancers16091746

**Published:** 2024-04-29

**Authors:** Ghalib Mobaraki, Shuai Shi, Kim M. Smits, Kim Severens, Kim Lommen, Dorit Rennspiess, Emil Chteinberg, Véronique Winnepenninckx, Iryna Samarska, Faisal Klufah, Axel zur Hausen

**Affiliations:** 1 Department of Pathology, GROW—Institute for Oncology & Reproduction, Maastricht University Medical Centre+, 6229 HX Maastricht, The Netherlands; ghalib.mobaraki@mumc.nl (G.M.); s.shuai@maastrichtuniversity.nl (S.S.); kim.smits@mumc.nl (K.M.S.); kim.severens@mumc.n (K.S.); k.lommen@maastrichtuniversity.nl (K.L.); dorit.rennspiess@mumc.nl (D.R.); v.winnepenninckx@mumc.nl (V.W.); iryna.samarska@mumc.nl (I.S.); fklufah@bu.edu.sa (F.K.); 2 Department of Laboratory Medicine, Faculty of Applied Medical Sciences, Jazan University, Jazan 45142, Saudi Arabia; 3 Institute of Human Genetics, Ulm University Medical Center, 89081 Ulm, Germany; 4 Department of Laboratory Medicine, Faculty of Applied Medical Sciences, Al-Baha University, Al Baha 65528, Saudi Arabia

**Keywords:** bovine meat milk factors (BMMFs), dietary cancer risk factors, etiopathogenesis, renal cell carcinoma (RCC), consensus polymerase chain reaction (PCR)

## Abstract

**Simple Summary:**

Circular bovine meat and milk factor (BMMF) DNAs have recently been identified in peritumoral tissues of human colon and breast cancers. Here we aimed to test the most common subtypes of renal cell carcinoma (RCC) for the prevalence of BMMF1 and BMMF2 DNA. We directly tested formalin-fixed and paraffin-embedded (FFPE) RCC and peritumoral tissues for BMMF1 and BMMF2 sequences by introducing novel consensus PCR primers. We demonstrate that BMMF1- and BMMF2- like DNA sequences can be reliably detected in FFPE tissues by consensus PCR. Our results demonstrate that BMMF1- and BMMF2- like sequences are frequently present in FFPE tissues of RCC and peritumoral tissues. Of interest, these sequences are more prevalent in peritumoral kidney tissues. These findings are potentially in line with the proposed model for BMMF-induced indirect colon carcinogenesis, which includes the presence of BMMFs in adjacent peritumoral tissues.

**Abstract:**

Previous studies have indicated a potential role of diet in the pathogenesis of renal cell carcinoma (RCC). Recently, circular bovine meat and milk factor (BMMF) DNAs have been identified in peritumoral tissues of human colon and breast cancers. Here, we investigated the prevalence of the DNA of these novel human pathogenic infectious agents in RCC and adjacent peritumoral renal tissues. DNA was extracted from formalin-fixed and paraffin-embedded (FFPE) RCC and peritumoral kidney tissues, including a test (n = 11) and a validation (n = 152) collection. BMMF1 and BMMF2 consensus primers were designed to screen for the presence of BMMF1- and BMMF2-like DNA. In addition, BMMF-specific PCR was performed on selected cases to test for the presence of additional regions of BMMF1 and BMMF2 genomes. A reference collection of hepatocellular carcinomas (HCCs; n = 60) and adjacent peritumoral liver tissues (n = 50) was also included. Our results demonstrated that BMMF1 and BMMF2 DNAs are frequently found in human RCC tissues and are particularly more prevalent in peritumoral kidney tissues. Of note, BMMF1 and BMMF2 genotype heterogeneity was higher in peritumoral kidney tissues compared to RCC tissues. This is the first study to directly test human FFPE tissues for BMMF1- and BMMF2-like DNA using consensus PCR and demonstrate BMMF DNA in neoplastic and peritumoral kidney tissues. The findings are in line with the recently proposed indirect etiopathogenetic role of BMMFs in, e.g., colorectal carcinogenesis. Follow-up studies are needed to explore the potential role of BMMFs in the etiopathogenesis of RCC.

## 1. Introduction

The recently discovered bovine meat and milk factors (BMMFs) have been described as infectious agents that in terms of ancestral origin are spanning between bacterial plasmids and single-stranded circular DNA viruses [1,2]. These replication-competent circular plasmid-like DNA sequences have been identified in and isolated from cow milk, dairy products, and bovine meat and are currently divided into four groups (BMMF1–BMMF4) according to their molecular characteristics [3,4]. It has been proposed that non-pathogenic persistent infections in animals and their products might be pathogenic if transmitted to humans [5]. BMMF DNAs have also been identified in the milk from water buffaloes, sheep, and goats, among others [6,7,8,9,10].

Based on the high degree of epidemiological concordance of colon and breast cancer incidence worldwide and the strong link of both cancers with the availability and consumption of meat and/or dairy products of bovine origin, BMMFs have been implicated in the etiology and pathogenesis of human colon and breast cancer [4,11,12]. Meanwhile, BMMF DNA and proteins have been detected in human colon cancer, i.e., in the interstitial macrophages of peritumoral tissues [13,14], as well as in lung and pancreatic cancer [15]. It has been postulated that through chronic inflammation, BMMFs presumably indirectly contribute to early colorectal carcinogenesis by the induction of oxidative stress and DNA mutations in adjacent replicating cells, progressively developing to colon cancer via known progenitors [12,16].

Epidemiological studies on diet and kidney cancer, of which renal cell carcinoma (RCC) is the most common type, have shown contradictory results. Inconsistent associations with nutrition in general and with meat and milk consumption in particular have been reported [17,18,19,20]. However, the geographic distribution of RCC incidence [11,21] also reveals a remarkable degree of epidemiological agreement with the geographic distribution of colon and breast cancer incidence, possibly pointing to a link with diet [1,2].

Kidney cancer accounts for approximately 2% of all cancers and ranks 16th among the most common human cancers [22]. It is estimated that each year, nearly 179,000 cancer-related deaths are due to kidney cancer, rendering it one of the most common causes of cancer-related deaths worldwide [22]. As approximately 70–80% of kidney cancer cases are renal cell carcinoma (RCC), RCC constitutes the most frequent type of kidney cancer [23,24]. Clear cell renal cell carcinoma (CCRCC) and papillary renal cell carcinoma (PRCC) are considered the most common subtypes of RCC [25]. RCC incidence has significantly increased in recent years and reveals significant geographical variation [11,21]. In the context of infectious pathogens, it is important to mention that immunosuppression in solid-organ-transplant recipients and obesity have been identified as risk factors for RCC [26,27,28,29]. Although many other risk factors for RCC have been identified and the increasing unraveling of the underlying molecular pathogenesis [30,31] has significantly contributed to our current understanding of RCC, the etiology of RCC yet remains obscure. Recently, it has been shown that exogenous BMMF DNA derived from milk or meat is able to replicate in human embryonic kidney (HEK293TT) cells [15].

In this study, we aimed to test the most common subtypes of RCC, i.e., CCRCC and PRCC, for the possible presence of BMMFs in formalin-fixed and paraffin-embedded (FFPE) RCC tissues [32,33]. For this purpose, we tested a set of well-characterized and freshly sampled RCC tissues using a highly sensitive novel broad-spectrum PCR for the presence of BMMF1- and BMMF2-like sequences. A large collection of RCC FFPE tissues was subsequently used to validate the findings of BMMF1- and BMMF2-like sequences.

## 2. Materials and Methods

### 2.1. Patients and Tissues

**Figure 1:**
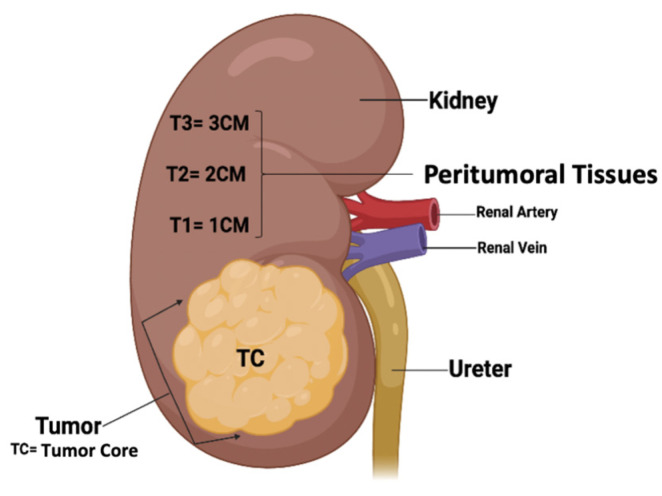
Diagram showing the grossing scheme and localization of RCC and peritumoral kidney tissues in the test collection. TC: tumor core; T1: 1 cm distance to the tumor; T2: 2 cm distance to the tumor; T3: 3 cm distance to the tumor (created in Biorender.com).

Two independent RCC collections were used in this study. The first collection, the test collection, included 11 RCCs (3 female and 8 males; mean age 71.6 years, range 43–85 years), of which 8 were CCRCC and 3 were PRCC, all collected from the Department of Pathology, MUMC+, Maastricht, The Netherlands (Table S1A). From each of the 11 RCCs, 4 FFPE tissue blocks were sampled from different locations (i.e., TC: tumor core; T1: 1 cm distance to tumor core, T2: 2 cm distance to tumor core, and T3: 3 cm distance to tumor core; see also Figure 1). In total, 44 FFPE tissue blocks were collected and analyzed.

The second collection, the validation collection, included 152 RCC FFPE tissues, as previously described [34]. This collection consisted of sporadic RCCs (92 female and 60 male patients; mean age 59.1 years, range 21–77 years). The RCCs (CCRCC: n = 135, 88.8%; PRCC: n = 17, 11.2%) were treated with radical or partial nephrectomy without any neo-adjuvant therapy (Table S1B) [34]. In addition, 39 peritumoral kidney tissue blocks were included (Table S1C).

An additional collection of 60 hepatocellular carcinoma (HCC) FFPE tissue blocks and 50 normal-liver FFPE tissue blocks (22 females and 38 males; mean age 66.3 years, range 28–84 years) was included as a reference collection at the Department of Pathology, MUMC+, Maastricht, The Netherlands, and tested for the presence of BMMF1- and BMMF2-like DNA. Clinico-pathological data are summarized in Table S2A,B.

The usage of patient material for this research project was approved by the Medical Ethics Review Committee of the Maastricht UMC+, The Netherlands (Ref no. 2021–2789), and the Ethics Committee Research UZ/KU Leuven, Belgium (Ref no. S62466, Amend-Id 0001).

### 2.2. DNA Extraction

DNA extraction was performed, as previously described [35]. In brief, 5 consecutive 10 µm thick sections were deparaffinized with xylene, subsequently lysed with proteinase K, and incubated overnight at 56 °C until complete tissue dissolution. DNA was extracted using the DNA Extraction and Purification Kit (Qiagen, Hilden, Germany). The quality and integrity of the purified DNA were assessed by spectrophotometry (Nanodrop 2000; Thermo Scientific, Wilmington, DE, USA) and by specimen control size (SCS) ladder DNA PCR (Figure S1). According to the assessment of all specimens by SCS ladder PCR [35], all specimens had sufficient DNA quality for further BMMF testing (Figure S1).

### 2.3. Polymerase Chain Reaction (PCR)

Broad-range PCR: To screen for the presence of BMMF1 and BMMF2 DNA, we designed two pairs of primers (see Table 1), each directed against a conserved region of BMMF1 (bps 158–363 (205 bps); reference: C1MI.3M.1, GenBank^®^ NCBI: LR21549) and of BMMF2 (bps 3–281 (279 bps); reference: C2MI.9As.2, GenBank^®^ NCBI: LR215600.1) [3]. The alignments of the DNA sequences of the respective BMMF1 and BMMF2 isolates used to design these broad-range primers are shown in Supplementary Figure S4A,B. PCRs were performed with 125 ng of genomic DNA using AmpliTaq Gold DNA polymerase (Applied Biosystems SimpliAmp Thermal Cycler; Thermo Fisher Scientific, Landsmeer, The Netherlands) in a final volume of 25 µL. PCR cycling conditions for consensus PCR were 7 min at 95 °C, followed by 45 cycles each at 94 °C for 1 min, 55 °C for 1 min, and 72 °C for 1 min, and a final elongation of 10 min at 72 °C.

To exclude potential contaminations and/or false-positive results, a synthetic BMMF1 (PCR product size: 247 bps) and BMMF2 (PCR product size: 321 bps) positive control was constructed (Eurofins Genomics, Germany) containing an additional 42 bp sequence (GTGGTGGTGGTGGTGGTGATGGTGGTGGTGGTGGTGGTGGTG for BMMF1 and GTGGTGGTGGTGGTGGTGGCTCCCGCTCCCGCTCCCGCTCCC for BMMF2). Negative controls were performed with PCR Master Mix using PCR-grade water, omitting template DNA. Moreover, DNA was isolated from tissue-free paraffin blocks and submitted to SCS PCR.

BMMF1- and BMMF2-specific PCR: Specific BMMF1 and BMMF2 primers were designed (Table 1) to assess whether other parts of the BMMF2 genome can be amplified in the FFPE tissues tested positive by BMMF2 consensus PCR. The same PCR conditions, as described before, were used except for the number of cycles (n = 40).

### 2.4. Sequence Analysis

PCR products were submitted to automated nucleotide sequencing in an ABI 3130XL genetic analyzer (ABI), and the obtained sequences were aligned with the reference sequences of BMMF1 (taxid: 2502151) and BMMF2 (taxid: 2502152) using the NCBI Entrez Nucleotide database, Basic Local Alignment Search Tool (https://blast.ncbi.nlm.nih.gov/Blast.cgi, accessed on 5 November 2022) [36]. Analyses were performed with multiple sequence alignments using the Clustal Omega algorithm (European Bioinformatics Institute, Ireland; https://www.ebi.ac.uk/Tools/msa/clustalo/, accessed on 5 November 2022) [37].

## 3. Results

**Figure 2:**
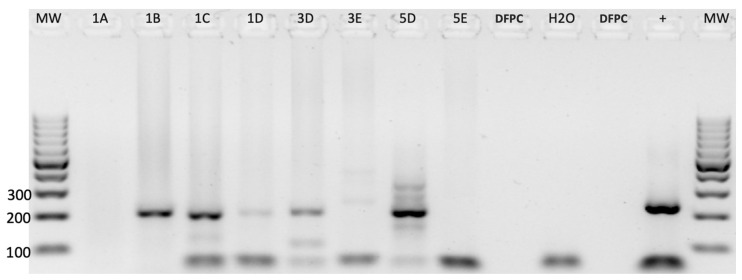
Consensus DNA PCRs in RCC (1B) and peritumoral kidney (1C, 1D, 3D, 5D) tissues of representative cases of the test collection for BMMF1 (205 bp). Abbreviations: H_2_O, water (non-template negative control); +, BMMF1, synthetically constructed positive controls (247 bp) (see Section 2); DFPC, DNA-free paraffin control; MW, molecular weight marker (Invitrogen™ by Thermo Fisher Scientific, Landsmeer, The Netherlands, 100 bp DNA ladder).

To test for the presence of BMMF1- and BMMF2-like DNA sequences by consensus PCR, we used an RCC collection (n = 11) as the test group. Resection specimens of eight CCRCC and three PRCC tissues were analyzed for the presence of BMMF1- and BMMF2-like DNA in the tumor core (TC) tissues and in tissues obtained from a distance of 1 (T1), 2 (T2), and 3 (T3) cm from the tumor core (see Figure 1). Both BMMF1- and BMMF2-like DNAs were found in 2 of 11 RCCs. However, in the adjacent peritumoral kidney tissue specimens (T1–T3), BMMF1-like DNA was found in 6 of 11 RCCs each, while BMMF2-like DNA was only found in 2 tissues (Table 2 and Table S1A, Figure 2 and Figure S2A,B). Analyzing the prevalence of BMMF1- and BMMF2-like DNA according to the two histological subtypes, i.e., CCRCC and PRCC, revealed that BMMF1 occurred in two of three patients with PRCC. However, BMMF2 DNA was restricted to patients with CCRCC (n = 2 (Table 2)).

We then used a retrospective specimen collection [31] of RCC FFPE tissues (n = 152) for validation purposes to detect BMMF1- and BMMF2-like DNA. Sequencing of the BMMF-PCR products detected BMMF1-like DNA in 6 (3.9%) of all RCC tumor tissues and BMMF2-like DNA sequences in 5 (3.2%) RCC tumor tissues (Table S1B, Figure S2A,B). Of the 39 peritumoral kidney tissues, BMMF1-like DNA sequences were found only in 1 specimen (2.6%); however, BMMF2-like DNA sequences were found in 9 (23%) of the 39 peritumoral kidney tissue specimens (Table S1C). Analyzing the prevalence of BMMF1 and BMMF2 DNA according to the two histological subtypes, i.e., CCRCC and PRCC, revealed that BMMF1- and BMMF2-like DNAs were restricted to CCRCC. No BMMF sequences were found in PRCCs in this collection.

Of note, only one CCRCC (no. 148, Table S1B) revealed positivity for both BMMF1 and BMMF2 DNA. In addition, one peritumoral kidney tissue (no. 35, Table S1C) showed double positivity for BMMF1 and BMMF2 DNA.

Due to the relatively frequent finding of BMMF1- and BMMF2-like DNA in RCC and the peritumoral kidney tissue specimens, we decided to test for the presence of further additional BMMF1 and BMMF2 sequence parts by using all different BMMF1- and BMMF2-specific primer pairs spanning the whole BMMF1 (C1MI.3M.1) (GenBank:LR215499.1) and BMMF2 (Sphinx 2.36) (GenBank: HQ444405.1) genomes (Figure 2 and Figure S3). We were, indeed, able to cover the full reference BMMF2 sequence by eight PCR amplicons and almost the entire BMMF1 reference sequence (seven amplicons detectable) in these peritumoral kidney tissues (see Table 1).

No significant correlations of BMMFs with any other clinico-pathological parameters were found. For example, BMMF1 was detected in 3/11 and BMMF2 was detected in 3/11 in male patients, while BMMF1 was detected in 2/11 and BMMF2 was detected in 1/11 in female patients (Table S1A,B).

Of interest, sequence analyses of the BMMF1 and BMMF2 PCR products in the retrospective RCC tissue collection revealed heterogeneous BMMF sequences, of which all except two (BMMF2) showed a relatively high degree of sequence identity to previously identified BMMFs (Table 3). Of note, two BMMF2-like DNA sequences had a sequence identity of only 75% or 83% (Table 3). In contrast, most BMMF2-like sequences of the peritumoral kidney tissue collection were of one BMMF2 genotype (i.e., GenBank HQ444405.1), and only one tissue specimen revealed a low sequence identity of 82% to a previously identified BMMF2 isolate (GenBank LR215600.1) (Table 3).

To evaluate whether our findings reflect a typical representation of BMMF1- and BMMF2-like DNA in human cancers, we assessed the presence of BMMF DNA in HCC specimens (n = 60). The etiology of HCC has been clearly linked to other risk factors, such as alcohol consumption and hepatitis viruses [38]. Of note, only one HCC specimen revealed BMMF1 DNA (1.6%), and no BMMF2 DNA was found in HCC tissues (0%). Of interest, adjacent peritumoral HCC tissues revealed 0/50 (0%) BMMF1 and only 1/50 (2%) BMMF2 DNA (Table S2A,B).

## 4. Discussion

In most parts of the Western world, bovine meat and milk are among the most popular food sources [17,18,19,20]. Retrospectively analyzing cancer prevalence, several recent studies have pointed to a significant correlation between the consumption of bovine meat and milk and the prevalence of colon, breast, pancreas, lung, and kidney cancer [1,2,15]. Single-stranded circular DNA has recently been isolated from dairy products, cow’s serum, and human tissues and has been classified as bovine meat and milk factors (BMMFs) [2,3,4]. Meanwhile, full-length BMMF DNAs have also been identified in the milk from water buffaloes, sheep, and goats [6,7]. It is assumed that BMMFs act as indirect carcinogens in the pathogenesis of, e.g., colon and breast cancer, as well as a variety of other chronic diseases associated with chronic inflammatory processes [2,13]. Moreover, a recent study has shown replication activity of BMMFs in human embryonic kidney (HEK293TT) cells by demonstrating the RNA transcription and protein expression of BMMF genes [15]. These data suggest that BMMFs can replicate in human tissues, possibly contributing to the onset or progression of colon, breast, and other cancers [39].

On the background of a possible epidemiological association between red meat consumption and RCC [17,18,19,20], we tested for the presence of BMMF DNA (BMMF1 and BMMF2) in RCC and some of its peritumoral kidney tissues. Indeed, we were able to reliably detect BMMF-like DNA in RCC FFPE tissues, proving that these consensus BMMF primers can be applied for BMMF1 and BMMF2 DNA-screening purposes in FFPE tissues. Worldwide, FFPE tissues represent the largest tissue resource for all types of cancers and other diseases. Hence, these novel consensus primers potentially represent a novel important tool in the assessment of BMMF-related diseases. The vast value and potential of consensus primers in the detection of infectious agents has been previously shown in the field of human papillomavirus (HPV) detection [40]. In this first study to screen FFPE tissues by consensus BMMF1 and BMMF2 PCR, BMMF sequences were more frequently detected in peritumoral kidney tissues compared to RCC tissues.

**Figure 3:**
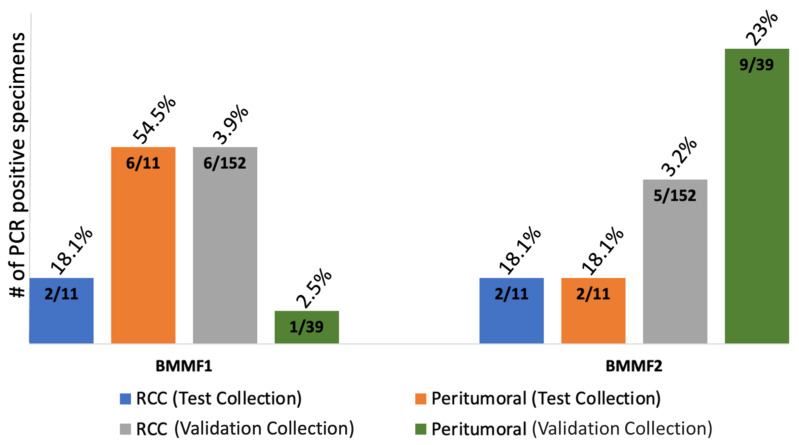
Summary of BMMF1 and BMMF2 PCR results for both RCC and peritumoral kidney tissue. Abbreviations: BMMF, bovine meat and milk factor; CCRCC, clear cell renal cell carcinoma.

It is highly interesting that BMMF-like DNA was more frequently found in peritumoral kidney tissues compared to RCC tissues in both collection groups (Figure 3). These findings are potentially in line with the proposed model for BMMF-induced indirect colon carcinogenesis, which includes the presence of BMMFs in adjacent peritumoral kidney tissues [2,3,13].

Of note, the BMMF genotypes identified in the peritumoral kidney tissues were quite homogeneous; however, the BMMF genotypes recognized in the RCC tissues were surprisingly heterogeneous (Table 2, Table 3 and Table 4). This BMMF genotype heterogeneity within RCC tissues could possibly reflect a superinfection (and therefore an epiphenomenon), e.g., related to reduced immune surveillance. However, this remains speculative, as also the possible interpretation that the peritumoral BMMF genotypes potentially reflect the tumorigenesis-relevant BMMFs for RCC development. The presence of more than one BMMF genotype in one tissue block was restricted to one RCC and one peritumoral kidney tissue, indicating that multiple BMMF genotype detection is possibly a rare event. The finding of some BMMF2 genotypes with only a low sequence identity of 75%, 82%, and 83% (Table 3) strongly suggests the presence of novel yet not-further-characterized BMMF genotypes.

Due to the detection of BMMF1 and BMMF2 DNA in RCC and peritumoral kidney FFPE tissues of the retrospective RCC collection, we also sought to amplify further additional parts of this specific BMMF1 and BMMF2 genotype. By using six to seven different BMMF1- and BMMF2-specific primer pairs covering the rest of the BMMF1 and BMMF2 genome, we were able to amplify all parts of the respected BMMF genome in the tested cases (Table 1 and Table 4; Figure S3). This approach confirmed the results of the BMMF1 and BMMF2 broad-range PCR and indirectly possibly suggests that the whole BMMF2 genome is present in these tissues.

The potential biological value of BMMF genotype identification and characterization in RCC and peritumoral kidney tissue seems to be underlined by the sparse finding of BMMF DNA in a large collection of HCC tissues and peritumoral liver tissues (Table S2A,B). Indeed, the etiology and pathogenesis of HCC have been clearly linked to other risk factors, such as alcohol consumption and hepatitis viruses [38]. However, BMMF1 and BMMF2 DNAs have previously been reported in colon, lung, and pancreatic cancer [2,13,14,15].

All previous studies testing for the presence of BMMF DNA have used a combination of rolling circle amplification (RCA) and subsequent BMMF DNA PCR. Of interest, two recent studies have reported the finding of BMMF DNA in an amazingly broad spectrum of food sources, possibly indicating that BMMFs are not solely of bovine origin and are widespread [8,9]. Although these findings await confirmation by others, the sheer finding of exogenous DNA in human tissues and body fluids, which is capable of replicating in HEK cells, is of significant concern. The presence, transcription, and translation of BMMFs in diverse human cancer tissues, in combination with potential widespread distribution in food, underline a possible role for BMMFs in human cancers [13,14,15].

We were able to demonstrate that a broad-range PCR approach for BMMF1- and BMMF2-like DNA detection is a suitable tool to screen large FFPE tissue collections in general and, here, RCC tissues in particular. Despite the successful detection of BMMF DNA, this study has some limitations. The use of retrospective FFPE tissues does restrict the amplification of BMMF DNA to smaller fragments of its genome. According to Figure S1, no DNA fragments larger than 400 bp could be reliably amplified from these tissues, which also hampers the further characterization or genotyping of the detected BMMF sequences. We regard this study as an explorative study. Future studies need to address whether the described BMMFs in RCC tissues are transcriptionally and translationally active and potentially relevant for the etiology or pathogenesis of RCC. In addition, it will be highly interesting to analyze the presence of BMMFs in RCC tissue in relation to, e.g., the VHL disease status, the presence of other mutations that are associated with RCC tumorigenesis, or paraneoplastic glomerulonephropathies. This research is currently ongoing in another large RCC study cohort.

## Figures and Tables

**Table 1:** Sequences of primers used for BMMF DNA PCR.

Oligonucleotide	Primer ID	Sequence (5′-3′)	Genome Location	Target	GenBank
**Consensus** **Primers**	BMMF1	FW: GAKGRCATWWRACRMSRYACCYAYCAATA RV: GATCCAAGTTGTAACTAGCGTTCATTAGG	158–363	BMMF1	LR215499.1
	BMMF2	FW: GGCAGATCAACACAGGGATAGAATWWCACG RV: CKWAHRSCWGCRCAVAWDGGRCANARYAAATGYYG	3–281	BMMF2	LR215600.1
**BMMF2-Specific Primers**	BMMF2-FA1	FW: CAGCATTTGCTATGTCCAATGTG RV: CCTGGTCAATCCGGTCAGT	180–531	BMMF2/Sphinx	HQ444405.1
	BMMF2-FA4	FW: ACTGACCGGATTGACCAGG RV: CCAAAAACGAAACGATAGAGCAG	513–833	BMMF2/Sphinx	HQ444405.1
	BMMF2-FA5	FW: TCGTTTTTGGTGAAAGGTC RV: TTCTCCAGTGGGAACAATTA	824–1147	BMMF2/Sphinx	HQ444405.1
	BMMF2-FA6	FW: CCCACTGGAGAACATTCTAT RV: TGCAAGAAATTAAGAATTGGTTAAAT	1136–1460	BMMF2/Sphinx	HQ444405.1
	BMMF2-FA7	FW: TGCAAACGGTTCAAAAAAGC RV: ATTGTTTCGTCGTCCAAAGA	1457–1781	BMMF2/Sphinx	HQ444405.1
	BMMF2-FA8	FW: GGACGACGAAACAATTAAAACTCT RV: ACAACATTTCGACCGATAGCC	1767–2103	BMMF2/Sphinx	HQ444405.1
	BMMF2-FA3	FW: GCTATCGGTCGAAATGTTGT RV: CCCTGTGTTGATCTGCATTA	2084–2333	BMMF2/Sphinx	HQ444405.1
**BMMF1-Specific Primers**	BMMF1-GM1	FW: CTAATGAACGCTAGTTACAACT RV: TGACCCAACGACTTGTAATAT	336–600	BMMF1/Sphinx	LR215499.1
	BMMF1-GM2	FW: TCGTTGGGTCAGCCAAATTGCTT RV: ATCCATTCGCTGATATTCAGTAT	590–857	BMMF1/Sphinx	LR215499.1
	BMMF1-GM3	FW: CAGCGAATGGATGTATTTAAACGT RV: AATACTGCCTAGTTTGCACAGAA	869–1115	BMMF1/Sphinx	LR215499.1
	BMMF1-GM4	FW: ACTAGGCAGTATTTCAGACTTGA RV: TTGCTTTTGGGGTTGAGGGGTTT	1103–1380	BMMF1/Sphinx	LR215499.1
	BMMF1-GM5	FW: CCCCAAAAGCAAAAACACTGTA RV: AAAACAAGCAAAAGCAACTATG	1369–1641	BMMF1/Sphinx	LR215499.1
	BMMF1-GM6	FW: TGCTTGTTTTCGGGTCTTAGGG RV: AAATGCCATCTGTATGCCTTGC	1632–1765	BMMF1/Sphinx	LR215499.1

Abbreviations: FW, forward; RV, reverse; n, number; cons, consensus; genome location is based on the sequence of BMMF1 and BMMF2.

**Table 2:** Summary of the PCR results of BMMF1 and BMMF2 consensus DNA PCR in the test collection (A) and of the sequencing results of BMMF1 and BMMF2 consensus PCR of the test collection (B).

(**A**)							
**Resection Sample**				**RCC Tissue**	**Peritumoral Kidney Tissue**		
**Patient#**		**Diagnosis**	**Clinical** **Stage**	**TC**	**T1** **(1 cm)**	**T2** **(2 cm)**	**T3** **(3 cm)**
**1**		CCRCC	1A	-	BMMF1	BMMF1	BMMF1
**2**		CCRCC	3A	-	BMMF2	-	BMMF1
**3**		PRCC	3A	BMMF1	BMMF1	BMMF1	-
**4**		CCRCC	3A	BMMF2	-	-	-
**5**		CCRCC	1A	-	-	BMMF1	-
**6**		PRCC	3A	BMMF1	-	BMMF1	-
**7**		PRCC	1B	-	-	-	-
**8**		CCRCC	1B	-	BMMF2	-	-
**9**		CCRCC	3A	BMMF2	-	-	-
**10**		CCRCC	1A	-	-	-	-
**11**		CCRCC	3A	-	BMMF1	-	-
(**B**)							
**BMMFs and Tissue**	**#**	**Sequence Results**				**Identity**	**GenBank**
**BMMF1** **RCC** **(d)**	**1**	BMMF1 DNA sequence, isolate C1MI.3M.1				99%	LR215499.1
	**2**	Uncultured bacterium plasmid clone HD4bpcirc putative replication protein gene				98%	KX838913.1
**BMMF2** **RCC** **(c)**	**1**	BMMF2 DNA sequence, isolate C2MI.9As.2				98%	LR215600.1
	**2**	BMMF2 DNA sequence, isolate C2MI.10As.1				98%	LR215597.1
**BMMF1** **peritumoral (b)**	**1**	Sphinx1.76-related DNA, HCBI3.108				98%	LK931495.1
	**2**	Uncultured bacterium plasmid clone HD4bpcirc putative replication protein gene				99%	KX838913.1
	**3**	Uncultured bacterium plasmid clone HD4bpcirc putative replication protein gene				98%	KX838913.1
	**4**	Uncultured bacterium plasmid clone HD4bpcirc putative replication protein gene				99%	KX838913.1
	**5**	Uncultured bacterium plasmid clone HD4bpcirc putative replication protein gene				99%	KX838913.1
	**6**	Uncultured bacterium plasmid clone HD4bpcirc putative replication protein gene				96%	KX838913.1
	**7**	Uncultured bacterium plasmid clone HD4bpcirc putative replication protein gene				95%	KX838913.1
	**8**	Uncultured bacterium plasmid clone HD4bpcirc putative replication protein gene				95%	KX838913.1
**BMMF2** **peritumoral (a)**	**1**	BMMF2 DNA sequence, isolate C2MI.9As.2				97%	LR215600.1
	**2**	BMMF2 DNA sequence, isolate C2MI.16B.11				95%	LR215580.1

According to Figure 1: TC: tumor core; T1: 1 cm distance to the tumor; T2: 2 cm distance to the tumor; T3: 3 cm distance to the tumor. -: no BMMF PCR product.

**Table 3:** Summary of the sequencing results of BMMF1 and BMMF2 consensus PCR in the validation collection.

BMMFs and Tissue#		Sequence Results	Identity	GenBank
BMMF1 RCC	1	Sphinx1.76-related DNA, replication-competent episomal DNA MSBI2.176	96%	LK931492.1
	2	Sphinx1.76-related DNA, replication-competent episomal DNA HCBI3.108	97%	LK931495.1
	3	BMMF1 DNA sequence, isolate C1MI.9M.1	94%	LR215496.1
	4	Sphinx1.76-related DNA, replication-competent episomal DNA HCBI3.108	100%	LK931495.1
	5	BMMF1 DNA sequence, isolate C1MI.15M.2	95%	LR215495.1
	6	Sphinx1.76-related DNA, replication-competent episomal DNA HCBI6.159	95%	LK931494.1
BMMF2 RCC	1	BMMF2 DNA sequence, isolate C2MI.15B.17	83%	LR215569.1
	2	TSE-associated circular DNA isolate Sphinx 2.36	98%	HQ444405.1
	3	BMMF2 DNA sequence isolate C2MI.9B.5	97%	LR215542.1
	4	BMMF2 DNA sequence, isolate C2MI.15B.1	92%	LR215553.1
	5	BMMF2 DNA sequence, isolate C2MI.8A.3	75%	LR215533.1
BMMF1 peritumoral	1	Sphinx1.76-related DNA, replication-competent episomal DNA HCBI3.108	98%	LK931495.1
BMMF2 peritumoral	1	BMMF2 DNA sequence, isolate C2MI.9As.2	82%	LR215600.1
	2	TSE-associated circular DNA isolate Sphinx 2.36, complete sequence	98%	HQ444405.1
	3	TSE-associated circular DNA isolate Sphinx 2.36, complete sequence	99%	HQ444405.1
	4	TSE-associated circular DNA isolate Sphinx 2.36, complete sequence	99%	HQ444405.1
	5	TSE-associated circular DNA isolate Sphinx 2.36, complete sequence	98%	HQ444405.1
	6	TSE-associated circular DNA isolate Sphinx 2.36, complete sequence	98%	HQ444405.1
	7	TSE-associated circular DNA isolate Sphinx 2.36, complete sequence	96%	HQ444405.1
	8	TSE-associated circular DNA isolate Sphinx 2.36, complete sequence	97%	HQ444405.1
	9	TSE-associated circular DNA isolate Sphinx 2.36, complete sequence	95%	HQ444405.1

Abbreviation: TSE, transmissible spongiform encephalopathy.

**Table 4:** Sequencing of 5 peritumoral kidney specimens of the validation collection screened with BMMF2/Sphinx2.36-specific primers.

Specific Primer	#	Sequence Results	Identity
**BMMF2-FA1**	**1**	TSE-associated circular DNA isolate Sphinx 2.36, complete sequence	99%
	**2**	TSE-associated circular DNA isolate Sphinx 2.36, complete sequence	99%
	**3**	TSE-associated circular DNA isolate Sphinx 2.36, complete sequence	99%
	**4**	TSE-associated circular DNA isolate Sphinx 2.36, complete sequence	99%
	**5**	TSE-associated circular DNA isolate Sphinx 2.36, complete sequence	99%
**BMMF2-FA4**	**1**	TSE-associated circular DNA isolate Sphinx 2.36, complete sequence	98%
	**2**	TSE-associated circular DNA isolate Sphinx 2.36, complete sequence	98%
	**3**	TSE-associated circular DNA isolate Sphinx 2.36, complete sequence	98%
	**4**	TSE-associated circular DNA isolate Sphinx 2.36, complete sequence	99%
	**5**	TSE-associated circular DNA isolate Sphinx 2.36, complete sequence	98%
**BMMF2-FA7**	**1**	TSE-associated circular DNA isolate Sphinx 2.36, complete sequence	99%
	**2**	TSE-associated circular DNA isolate Sphinx 2.36, complete sequence	98%
	**3**	TSE-associated circular DNA isolate Sphinx 2.36, complete sequence	99%
	**4**	TSE-associated circular DNA isolate Sphinx 2.36, complete sequence	98%
	**5**	TSE-associated circular DNA isolate Sphinx 2.36, complete sequence	98%
**BMMF2-FA8**	**1**	TSE-associated circular DNA isolate Sphinx 2.36, complete sequence	99%
	**2**	TSE-associated circular DNA isolate Sphinx 2.36, complete sequence	99%
	**3**	TSE-associated circular DNA isolate Sphinx 2.36, complete sequence	99%
	**4**	TSE-associated circular DNA isolate Sphinx 2.36, complete sequence	99%
	**5**	TSE-associated circular DNA isolate Sphinx 2.36, complete sequence	99%

## Data Availability

The original contributions presented in the study are included in the article/Supplementary Materials, further inquiries can be directed to the corresponding author.

## Supplementary Materials

The following supporting information can be downloaded from https://www.mdpi.com/article/10.3390/cancers16091746/s1: Figure S1: DNA quality assessment by specimen control size ladder; Figure S2: Schematic illustration of entire BMMF DNA sequences aligned with consensus primers and sequence results; Figure S3: Schematic illustration of BMMF1/Sphinx and BMMF2/Sphinx entire genome characterization in RCC and peritumoral tissues; Figure S4: Sequence alignments of BMMF1 and BMMF2 conserved regions. Table S1: Clinicopathological data and consensus BMMF1 and 2 DNA PCR results of the RCC Collections; Table S2: Clinicopathological data and consensus BMMF1 and 2 DNA PCR results of the Hepatocellular Carcinoma (HCC) Control Collection and peritumoral liver tissues.
