# Supplementary material for: Bovine Meat and Milk Factor-like Sequences Are Frequently Detected in Renal Cell Carcinoma Tissues
Source: Cancers (Basel). 2024 Apr 29;16(9):1746. doi: 10.3390/cancers16091746 (PMC11083248; doi:10.3390/cancers16091746)
Supplement: Supplementary file 1 [file cancers-16-01746-s001.zip › cancers-2943479-supplementary.pdf]

Legends to the supplementary tables

Table S 1A: Clinicopathological data and consensus BMMF1 and 2 DNA PCR results of the RCC Test Collection

Sample ID*	Tissue Loc.	Gender	Age	Diagnosis	Clinical Stage	BMMF1	BMMF2
1A	TC	M	62	CCRCC	1A	-	-
1B	T1	M	62	CCRCC	1A	+	-
1C	T2	M	62	CCRCC	1A	+	-
1D	T3	M	62	CCRCC	1A	+	-
2D	TC	M	70	CCRCC	3A	-	-
2A	T1	M	70	CCRCC	3A	-	+
2E	T2	M	70	CCRCC	3A	-	-
2B	T3	M	70	CCRCC	3A	+	-
3A	TC	F	75	PRCC	3A	+	-
3C	T1	F	75	PRCC	3A	+	-
3D	T2	F	75	PRCC	3A	+	-
3E	T3	F	75	PRCC	3A	-	-
4C	TC	M	73	CCRCC	3A	-	+
4B	T1	M	73	CCRCC	3A	-	-
4D	T2	M	73	CCRCC	3A	-	-
4E	T3	M	73	CCRCC	3A	-	-
5A	TC	F	69	CCRCC	1A	-	-
5C	T1	F	69	CCRCC	1A	-	-
5D	T2	F	69	CCRCC	1A	+	-
5E	T3	F	69	CCRCC	1A	-	-
6A	TC	M	77	PRCC	3A	+	-
6C	T1	M	77	PRCC	3A	-	-
6D	T2	M	77	PRCC	3A	+	-
6E	T3	M	77	PRCC	3A	-	-
7E	TC	M	73	PRCC	1B	-	-
7C	T1	M	73	PRCC	1B	-	-
7B	T2	M	73	PRCC	1B	-	-
7A	T3	M	73	PRCC	1B	-	-
8E	TC	M	43	CCRCC	1B	-	-
8C	T1	M	43	CCRCC	1B	-	+
8B	T2	M	43	CCRCC	1B	-	-
8A	T3	M	43	CCRCC	1B	-	-
9A	TC	F	77	CCRCC	3A	-	+
9C	T1	F	77	CCRCC	3A	-	-
9D	T2	F	77	CCRCC	3A	-	-
9E	T3	F	77	CCRCC	3A	-	-
10A	TC	M	84	CCRCC	1A	-	-
10C	T1	M	84	CCRCC	1A	-	-
10D	T2	M	84	CCRCC	1A	-	-
10E	T3	M	84	CCRCC	1A	-	-
11A	TC	M	85	CCRCC	3A	-	-
11D	T1	M	85	CCRCC	3A	+	-
11C	T2	M	85	CCRCC	3A	-	-
11E	T3	M	85	CCRCC	3A	-	-

M, Male; F, Female; CCRCC, Clear Cell Renal Cell Carcinoma; PRCC, Papillary Renal Cell Carcinoma; BMMF, Bovine Meat and Milk Factor; Tissue Loc, Tissue Localisation (TC, T1, T2, T3) according to Fig 1A; +, positive (as confirmed by sequencing); −, negative; *, Samples were tested as blind choice.

Table S 1B: Clinicopathological data and consensus BMMF1 and 2 DNA PCR results of the RCC Validation Collection

Lab#	Age	Gender	Diagnosis	Clinical Stage	BMMF1	BMMF2	Lab #	Age	Gender	Diagnosis	Clinical Stage	BMMF1	BMMF2
1	33	F	CCRCC	3	-	-	77	**63**	**F**	CCRCC	**2**	**-**	**-**
2	69	M	CCRCC	2	-	-	78	62	F	CCRCC	2	-	-
3	73	M	CCRCC	2	-	-	79	59	M	CCRCC	3	-	-
4	70	F	CCRCC	2	-	-	80	53	F	CCRCC	3	-	-
5	61	F	CCRCC	2	-	-	81	36	F	CCRCC	2	+	-
6	64	M	CCRCC	3	-	-	82	68	F	CCRCC	2	-	+
7	31	M	CCRCC	2	-	-	83	52	M	CCRCC	1	-	-
8	67	F	CCRCC	2	-	-	84	28	M	CCRCC	2	-	-
9	70	F	CCRCC	2	-	-	85	66	F	PRCC	4	-	-
10	71	M	CCRCC	2	+	-	86	55	M	CCRCC	2	-	-
11	68	F	CCRCC	2	-	-	87	69	F	CCRCC	3	-	-
12	59	F	CCRCC	3	-	-	88	71	F	CCRCC	2	-	-
13	72	F	CCRCC	2	-	-	89	60	F	CCRCC	2	-	-
14	64	M	CCRCC	3	-	+	90	69	F	CCRCC	3	-	-
15	69	F	CCRCC	4	-	-	91	49	M	CCRCC	2	-	+
16	62	F	PRCC	4	-	-	92	61	M	PRCC	2	-	-
17	40	M	CCRCC	2	-	-	93	77	F	CCRCC	2	-	+
18	57	M	CCRCC	3	-	-	94	46	F	CCRCC	4	-	-
19	71	F	CCRCC	3	+	-	95	70	M	CCRCC	2	-	-
20	73	M	CCRCC	2	-	-	96	55	M	CCRCC	4	-	-
21	69	M	CCRCC	2	-	-	97	60	M	CCRCC	2	-	-
22	65	F	CCRCC	3	-	-	98	50	F	CCRCC	2	-	-
23	57	F	CCRCC	3	-	-	99	36	F	CCRCC	3	-	-
24	75	M	PRCC	2	-	-	100	49	F	CCRCC	2	-	-
25	73	F	CCRCC	3	-	-	101	70	M	CCRCC	4	-	-
26	53	M	CCRCC	2	-	-	102	49	M	CCRCC	3	-	-
27	53	F	CCRCC	3	-	-	103	75	F	CCRCC	2	-	-
28	46	F	CCRCC	4	-	-	104	75	F	PRCC	2	-	-
29	67	F	CCRCC	4	-	-	105	67	M	CCRCC	2	-	-
30	47	F	CCRCC	4	-	-	106	60	F	CCRCC	2	-	-
31	70	M	CCRCC	4	-	-	107	70	M	CCRCC	2	-	-
32	58	F	CCRCC	3	-	-	108	51	M	CCRCC	2	-	-
33	55	F	CCRCC	3	-	-	109	43	F	CCRCC	3	-	-
34	48	F	CCRCC	2	-	-	110	65	M	CCRCC	4	-	-
35	48	F	PRCC	1	-	-	111	49	F	CCRCC	2	-	-
36	64	F	CCRCC	2	-	-	112	60	F	CCRCC	4	-	-
37	75	F	CCRCC	3	-	-	113	62	F	CCRCC	2	-	-
38	64	F	CCRCC	2	-	-	114	45	M	CCRCC	3	-	-
39	45	F	PRCC	2	-	-	115	**65**	**F**	**CCRCC**	**2**	**-**	**-**
40	54	F	CCRCC	2	-	-	116	65	F	PRCC	3	-	-
41	59	F	CCRCC	2	-	-	117	53	M	CCRCC	2	-	-
42	66	F	CCRCC	2	-	-	118	71	M	CCRCC	2	-	-
43	57	F	CCRCC	2	-	-	119	60	M	CCRCC	2	-	-
44	73	F	CCRCC	2	-	-	120	67	F	CCRCC	3	-	-
45	50	F	CCRCC	2	-	-	121	60	M	PRCC	1	-	-
46	76	M	CCRCC	2	-	-	122	68	M	CCRCC	2	-	-
47	74	F	CCRCC	2	+	-	123	59	M	CCRCC	4	-	-
48	69	F	CCRCC	4	-	-	124	67	F	PRCC	3	-	-
49	63	M	CCRCC	2	-	-	125	71	F	CCRCC	2	-	-
50	74	M	CCRCC	2	+	-	126	51	M	CCRCC	2	-	-

51	59	F	CCRCC	1	-	-	127	67	F	CCRC	2	-	-
52	53	M	CCRCC	2	-	-	128	76	M	CCRCC	2	-	-
53	76	F	CCRCC	3	-	-	129	46	F	CCRCC	2	-	-
54	58	F	PRCC	2	-	-	130	66	M	PRCC	1	-	-
55	74	M	CCRCC	4	-	-	131	60	F	CCRCC	4	-	-
56	26	F	CCRCC	2	-	-	132	49	M	CCRCC	3	-	-
57	58	F	CCRCC	2	-	-	133	63	M	PRCC	2	-	-
58	45	F	CCRCC	2	-	-	134	51	M	CCRCC	2	-	-
59	37	M	CCRCC	4	-	-	135	55	M	CCRCC	3	-	-
60	48	F	CCRCC	4	-	-	136	70	M	CCRCC	2	-	-
61	21	M	CCRCC	4	-	-	137	68	F	PRCC	2	-	-
62	72	F	PRCC	3	-	-	138	73	F	CCRCC	1	-	-
63	70	M	CCRCC	2	-	-	139	52	F	CCRCC	4	-	-
64	67	M	CCRCC	2	-	-	140	65	F	CCRCC	3	-	-
65	60	F	CCRCC	2	-	-	141	72	F	CCRCC	2	-	-
66	63	M	CCRCC	3	-	-	142	62	M	CCRCC	2	-	-
67	63	F	CCRCC	4	-	-	143	52	M	CCRCC	3	-	-
68	42	F	CCRCC	2	-	-	144	34	F	CCRCC	2	-	-
69	69	F	CCRCC	4	-	-	145	62	M	CCRCC	2	-	-
70	77	F	CCRCC	2	-	-	146	43	M	CCRCC	2	-	-
71	56	F	CCRCC	2	-	-	147	66	M	CCRCC	2	-	-
72	67	F	CCRCC	2	-	-	148	68	F	CCRCC	2	+	+
73	69	F	PRCC	4	-	-	149	73	F	PRCC	2	-	-
74	47	F	CCRCC	2	-	-	150	49	F	CCRCC	2	-	-
75	56	F	CCRCC	4	-	-	151	55	F	CCRCC	2	-	-
76	67	F	CCRCC	2	-	-	152	72	F	CCRCC	4	-	-

M, Male, F, Female; CCRCC, Clear Cell Renal Cell Carcinoma; PRCC, Papillary Renal Cell Carcinoma; BMMF, Bovine Meat and Milk Factor; +, positive (as confirmed by sequencing); −, negative.

Table S 1C: Clinicopathological data and consensus BMMF1 and 2 DNA PCR results of the peritumoral kidney tissues of the Validation Collection

**Lab#**	**Age**	**Gender**	**Diagnosis**	**Clinical Stage**	**BMMF1**	**BMMF2**	**Lab#**	**Age**	**Gender**	**Diagnosis**	**Clinical Stage**	**BMMF1**	**BMMF2**
**1**	63	M	CCRCC	2	-	-	**22**	59	F	CCRCC	2	-	-
**2**	50	M	CCRCC	2	-	-	**23**	62	M	CCRCC	2	-	-
**3**	34	F	CCRCC	3	-	-	**24**	33	F	CCRCC	3	-	-
**4**	45	M	CCRCC	2	-	-	**25**	49	F	CCRCC	3	-	-
**5**	60	M	CCRCC	2	-	-	**26**	37	M	CCRCC	4	-	-
**6**	59	F	CCRCC	2	-	-	**27**	58	F	CCRCC	2	-	+
**7**	54	M	CCRCC	2	-	-	**28**	55	F	CCRCC	2	-	-
**8**	46	F	CCRCC	2	-	-	**29**	48	F	CCRCC	4	-	-
**9**	50	M	CCRCC	2	-	-	**30**	56	F	CCRCC	2	-	+
**10**	61	M	CCRCC	2	-	-	**31**	47	M	CCRCC	2	-	+
**11**	63	F	CCRCC	4	-	-	**32**	40	M	CCRCC	2	-	+
**12**	42	F	CCRCC	2	-	-	**33**	50	F	CCRCC	3	-	+
**13**	36	F	CCRCC	2	-	-	**34**	55	M	CCRCC	3	-	+
**14**	67	F	CCRCC	2	-	-	**35**	43	F	CCRCC	2	+	+
**15**	74	M	CCRCC	2	-	-	**36**	52	M	CCRCC	4	-	+
**16**	56	F	CCRCC	2	-	-	**37**	35	M	CCRCC	2	-	+
**17**	72	M	CCRCC	3	-	-	**38**	63	M	CCRCC	2	-	-

**18**	60	M	CCRCC	2	-	-	**39**	53	M	CCRCC	2	-	-
**19**	69	F	CCRCC	4	-	-							
**20**	53	M	CCRCC	2	-	-							
**21**	77	F	CCRCC	4	-	-							

M, Male; F, Female, CCRCC, Clear Cell Renal Cell Carcinoma; PRCC, Papillary Renal Cell Carcinoma; BMMF, Bovine Meat and Milk Factor; +, positive (as confirmed by sequencing); −, negative.

Supplementary tables

Table S 2A: Clinicopathological data and consensus BMMF1 and 2 DNA PCR results of the Hepatocellular Carcinoma (HCC) Control Collection

Lab ID	Gender	Age	Diagnosis	Clinical Stage	BMMF1	BMMF2	Lab ID	Gender	Age	Diagnosis	Clinical Stage	BMMF1	BMMF2
**HCC1**	M	77	HCC NOS	II	-	-	HCC31	M	76	HCC NOS	IA	-	-
**HCC2**	F	48	HCC NOS	III	-	-	HCC32	M	49	HCC NOS	IA	-	-
**HCC3**	M	46	HCC NOS	II	-	-	HCC33	F	81	HCC NOS	III	-	-
**HCC4**	M	61	HCC NOS	II	-	-	HCC34	M	80	HCC NOS	III	-	-
**HCC5**	F	48	HCC NOS	III	-	-	HCC35	M	75	fibrolamellar	III	-	-
**HCC6**	F	58	HCC NOS	III	-	-	HCC36	M	64	HCC NOS	II	-	-
**HCC7**	F	28	HCC NOS	IB	-	-	HCC37	F	65	Clear cell	II	-	-
**HCC8**	M	55	HCC NOS	IB	-	-	HCC38	F	57	HCC NOS	IA	-	-
**HCC9**	M	55	HCC NOS	III	-	-	HCC39	F	76	HCC NOS	III	-	-
**HCC10**	M	62	HCC NOS	III	-	-	HCC40	F	56	HCC NOS	II	-	-
**HCC11**	M	80	HCC NOS	III	+	-	HCC41	M	69	HCC NOS	III	-	-
**HCC12**	F	63	HCC NOS	II	-	-	HCC42	F	72	Steatohepatic	II	-	-
**HCC13**	F	61	HCC NOS	IB	-	-	HCC43	M	60	HCC NOS	II	-	-
**HCC14**	M	77	HCC NOS	III	-	-	HCC44	M	60	HCC NOS	IB	-	-
**HCC15**	F	69	HCC NOS	II	-	-	HCC45	M	77	HCC NOS	III	-	-
**HCC16**	M	68	HCC NOS	IA	-	-	HCC46	M	74	HCC NOS	II	-	-
**HCC17**	M	73	fibrolamellar	IA	-	-	HCC47	M	74	Steatohepatic	II	-	-
**HCC18**	M	71	Clear cell	IA	-	-	HCC48	M	75	HCC NOS	II	-	-
**HCC19**	F	59	HCC NOS	III	-	-	HCC49	F	62	HCC NOS	IB	-	-
**HCC20**	F	63	Clear cell	II	-	-	HCC50	F	84	HCC NOS	II	-	-
**HCC21**	F	60	HCC NOS	II	-	-	HCC51	M	63	HCC NOS	II	-	-
**HCC22**	F	70	HCC NOS	III	-	-	HCC52	M	75	Steatohepatic	II	-	-
**HCC23**	M	73	HCC NOS	IA	-	-	HCC53	F	62	HCC NOS	II	-	-
**HCC24**	M	77	HCC NOS	III	-	-	HCC54	M	64	HCC NOS	IV	-	-
**HCC25**	M	74	steatohepatic	III	-	-	HCC55	F	58	HCC NOS	II	-	-
**HCC26**	M	50	HCC NOS	IV	-	-	HCC56	M	63	HCC NOS	IA	-	-
**HCC27**	M	65	HCC NOS	IA	-	-	HCC57	M	73	Steatohepatic	II	-	-
**HCC28**	M	81	HCC NOS	III	-	-	HCC58	M	77	HCC NOS	III	-	-
**HCC29**	F	69	HCC NOS	III	-	-	HCC59	F	84	HCC NOS	III	-	-
**HCC30**	M	66	steatohepatic	III	-	-	HCC60	M	63	Clear cell	II	-	-

NOS, Neoplasm; M, Male; F, Female, HCC, Hepatocellular Carcinoma; BMMF, Bovine Meat and Milk Factor; +, positive (as confirmed by sequencing); −, negative.

Table S 2B: Clinicopathological data and consensus BMMF1 and 2 DNA PCR results of the corresponding peritumoral liver tissues of the HCC Control Collection (see table S 2A)

Lab ID	Gender	Age	Diagnosis	Clinical Stage	BMMF1	BMMF2	Lab ID	Gender	Age	Diagnosis	Clinical Stage	BMMF1	BMMF2
**1**	M	77	HCC NOS	II	-	-	**26**	M	80	HCC NOS	IA	-	-
**2**	F	48	HCC NOS	III	-	-	**27**	M	75	HCC NOS	IA	-	-
**3**	M	61	HCC NOS	II	-	-	**28**	M	64	HCC NOS	III	-	-
**4**	M	48	HCC NOS	II	-	-	**29**	F	65	HCC NOS	III	-	-
**5**	F	58	HCC NOS	III	-	-	**30**	F	75	fibrolamellar	III	-	-
**6**	F	28	HCC NOS	III	-	-	**31**	F	76	HCC NOS	II	-	-
**7**	F	55	HCC NOS	IB	-	-	**32**	F	65	Clear cell	II	-	-
**8**	M	55	HCC NOS	IB	-	-	**33**	M	69	HCC NOS	IA	-	-
**9**	M	62	HCC NOS	III	-	-	**34**	F	72	HCC NOS	III	-	-
**10**	M	61	HCC NOS	III	-	-	**35**	M	60	HCC NOS	II	-	-
**11**	M	77	HCC NOS	III	-	-	**36**	M	77	HCC NOS	III	-	-
**12**	F	68	HCC NOS	II	-	-	**37**	M	74	steatohepatic	II	-	-
**13**	F	73	HCC NOS	IB	-	-	**38**	M	74	HCC NOS	II	-	-
**14**	M	71	HCC NOS	III	-	-	**39**	M	75	HCC NOS	IB	-	-
**15**	F	59	HCC NOS	II	-	-	**40**	F	62	HCC NOS	III	-	-
**16**	M	60	HCC NOS	IA	-	-	**41**	F	84	HCC NOS	II	-	-
**17**	M	73	fibrolamellar	IA	-	-	**42**	M	75	steatohepatic	II	-	-
**18**	M	71	Clear cell	IA	-	-	**43**	M	75	HCC NOS	II	-	-
**19**	F	50	HCC NOS	III	-	-	**44**	F	62	HCC NOS	IB	-	-
**20**	F	63	Clear cell	II	-	-	**45**	M	64	HCC NOS	II	-	-
**21**	F	81	HCC NOS	II	-	-	**46**	F	58	HCC NOS	II	-	-
**22**	F	69	HCC NOS	III	-	+	**47**	M	73	steatohepatic	II	-	-
**23**	M	76	HCC NOS	IA	-	-	**48**	M	63	HCC NOS	II	-	-
**24**	M	49	HCC NOS	III	-	-	**49**	M	77	HCC NOS	IV	-	-
**25**	M	74	steatohepatic	III	-	-	**50**	M	63	HCC NOS	II	-	-

M, Male, F, Female, HCC, Hepatocellular Carcinoma; BMMF, Bovine Meat and Milk; +, positive (as confirmed by sequencing); −, negative.

Figure S1:

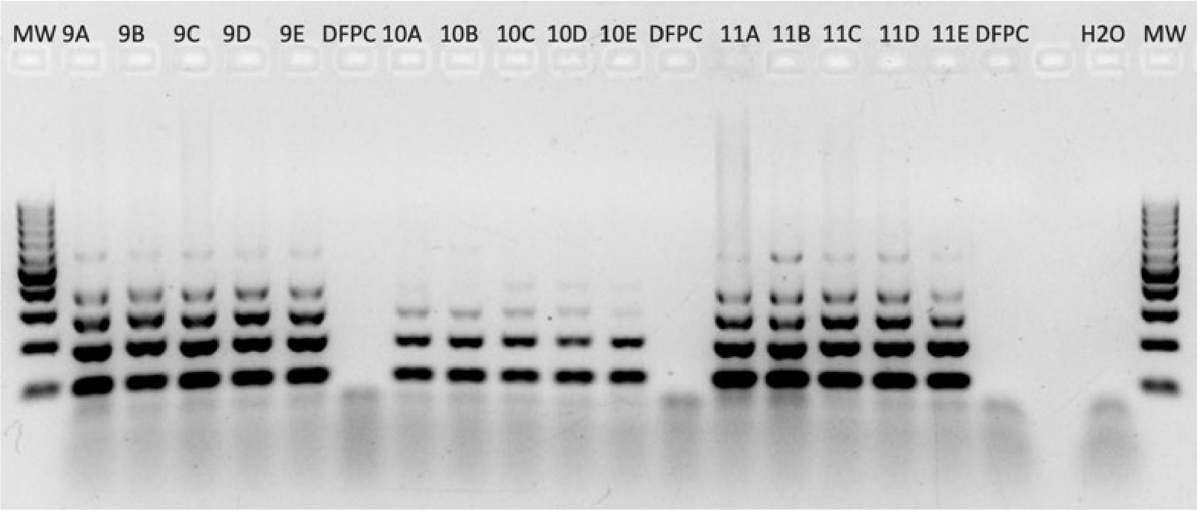

Fig. S1 Agarose gel showing the specimen control size (SCS) ladder protocol to validate the DNA quality to be eligible for PCR testing. Abbreviations: H2O water non-template negative control; DFPC, DNA-free paraffin control; MW, molecular weight marker (Invitrogen™, 100bp DNA ladder)

Figure S2 :

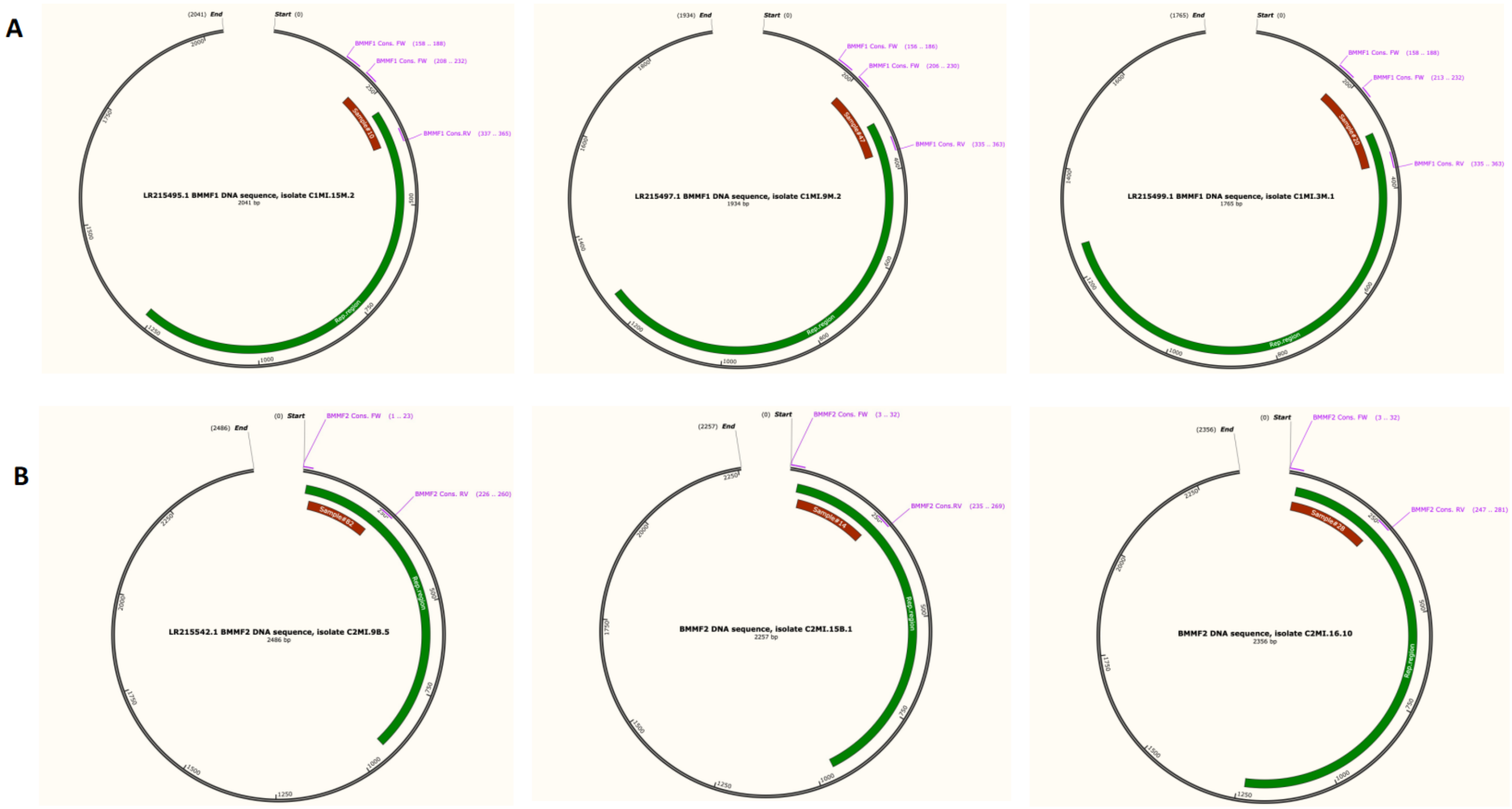

Fig. S2 Schematic illustration of entire BMMF DNA aligned with consensus primers and sequence result examples of findings in RCC collections. Replication region (green); our sequence result (red). (A) BMMF1, samples no.10, 47 from RCC of validation and no. 20 from test collection, and (B) BMMF2, samples no. 82, 14 from RCC of validation and no. 28 from test collection (Created with SnapGene®)

Figure S3 :

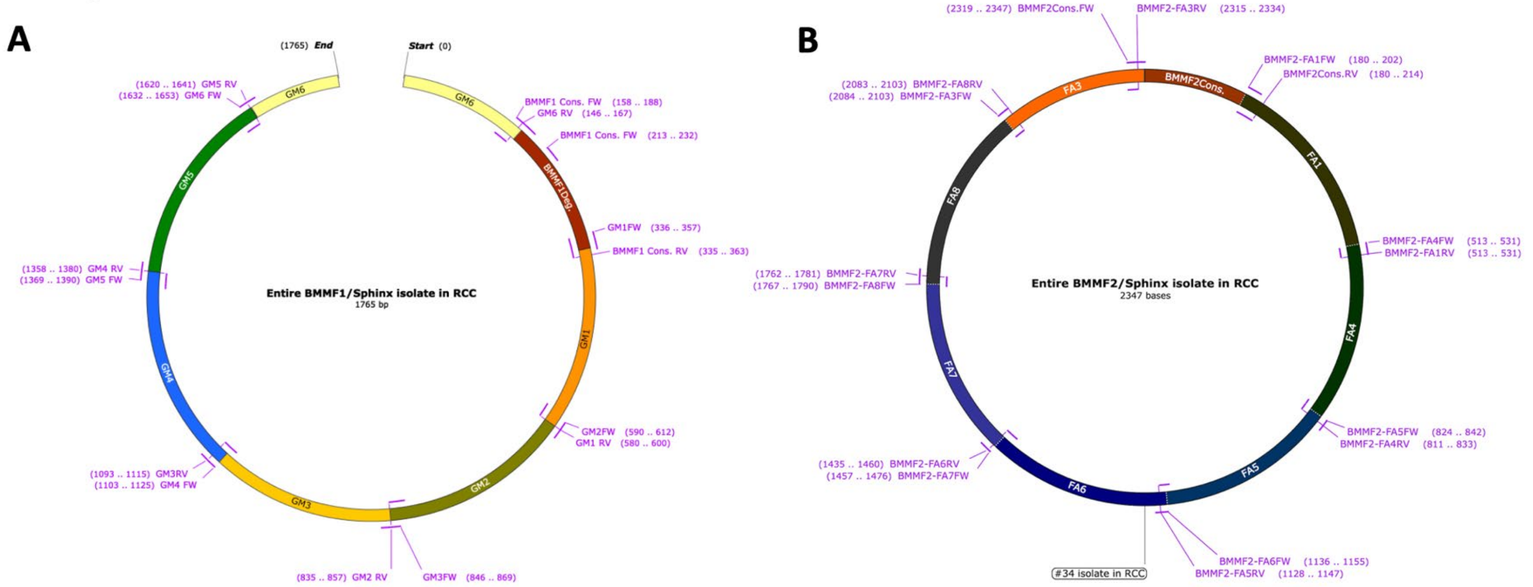

Fig. S3 Schematic illustration of BMMF1/Sphinx and BMMF2/Sphinx entire genome characterization in RCC and peritumoral tissues of validation collection respectively. (A) Sample no. 47 and (B) Sample no. 34 (Created with SnapGene®)

Figure S4 A:

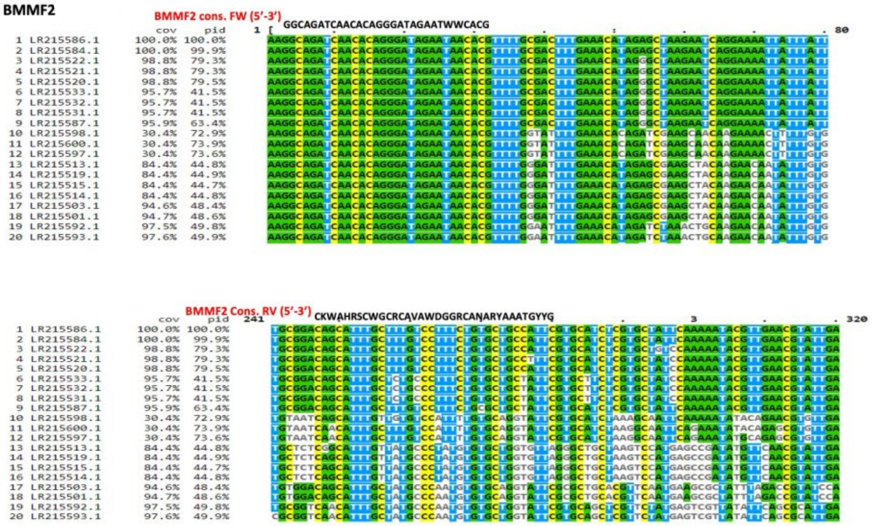

Fig. S4 Sequence alignments of BMMF1 (A) and BMMF2 (B) conserved regions of the most common BMMFs which can be detected with the consensus primers; cov, coverage; pid, percent identity (designed by the Clustal Omega algorithm by The European Bioinformatics Institute).

Figure S4 B:

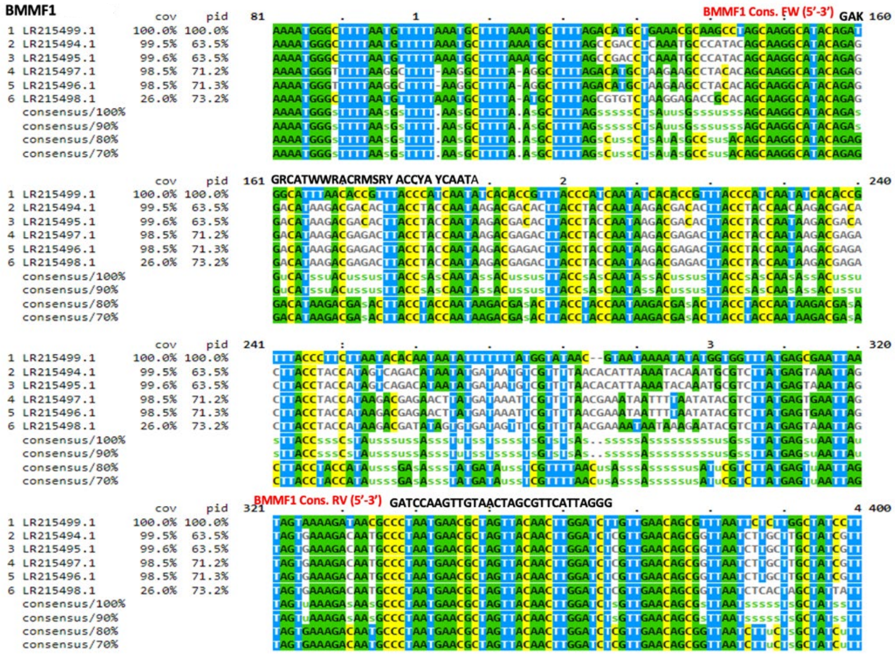
BMMF1
BMMF1 Cons. FW (5'-3')
GAK
GRCATWWRACRMSRY ACCYA YCAATA
BMMF1 Cons. RV (5'-3')
GATCCAAGTTGTAACTAGCGTTCATTAGGG
cov
pid
1 LR215499.1 100.0% 100.0%
2 LR215494.1 99.5% 63.5%
3 LR215495.1 99.6% 63.5%
4 LR215497.1 98.5% 71.2%
5 LR215496.1 98.5% 71.3%
6 LR215498.1 26.0% 73.2%
consensus/100%
consensus/90%
consensus/80%
consensus/70%
81
160
161
240
241
320
321
400
